# Supplementary material for: Analysis of PPARγ Signaling Activity in Psoriasis
Source: Int J Mol Sci. 2021 Aug 10;22(16):8603. doi: 10.3390/ijms22168603 (PMC8395241; doi:10.3390/ijms22168603)
Supplement: Supplementary file 1 [file ijms-22-08603-s001.zip › Supplemental materials_Analysis of PPARg signaling activity in psoriasis/Pathway models/Models images and html files/Model of PPARG related pathways in psoriasis (short version)/10208.html]

retinoid-X receptor subfamily


# Functional Class retinoid-X receptor subfamily

|  |  |
| --- | --- |
| URN | urn:agi-go:0004886 |
| Total Entities | 3 |
| Connectivity | 2601 |
| Name | retinoid-X receptor subfamily |

---

|  |  |
| --- | --- |
| ChildConcepts | RXRA |
|  | RXRB |
|  | RXRG |

---

|  |  |
| --- | --- |
| Pathway | Thyroid Hormones Common Genomic Effects in Hyperthyroidism |
|  | AHR Signaling in Treg and Dendritic Cells Function |
|  | Lipids Enhance Apoptotic Cell Engulfment and Reduce Inflammation |
|  | Retinoic Acid in Meiosis Regulation |
|  | Retinoic Acid Signaling Inhibition in Pre-Puberty Block of Meiosis |
|  | Vitamin D Activates Transcription |
|  | Vitamin D Represses Transcription |
|  | Bone Remodeling in Hyperthyroidism |
|  | Cardiovascular Effects in Hyperthyroidism |
|  | Thyroid Hormones in Adipose Tissue Metabolism |
|  | Neighbors of retinoid-X receptor subfamily |
|  | Model of PPARG related pathways in psoriasis (short version) |
|  | PPARs and neuroinflamation |
|  | 2\_3\_Effects of thyroid hormone deficiency: levothyroxine systemic effects |
|  | 2\_1\_General pathways of thyroid hormone signaling |
|  | 2\_2\_Effects of thyroid hormone deficiency: cell-specific effects of thyroid hormone action |
|  | before laser treatment |
|  | PPARG signaling after laser treatment |

---

|  |  |
| --- | --- |
| MedScan ID | 12104886 |

---

|  |  |
| --- | --- |
| Alias | retinoic X receptors |
|  | retinoid-X-receptor for 9-cis-retinoic acid |
|  | retinoid-X receptor |
|  | RXR |
|  | retinoic X receptor |
|  | retinoid-X receptors |
|  | RXRs |

---

|  |  |
| --- | --- |
| GO ID | 0004886 |

---
